# Structural and Functional Analysis of the DEAF-1 and BS69 MYND Domains

**DOI:** 10.1371/journal.pone.0054715

**Published:** 2013-01-25

**Authors:** Fatiha Kateb, Helene Perrin, Konstantinos Tripsianes, Peijian Zou, Roberta Spadaccini, Matthew Bottomley, Titus M. Franzmann, Johannes Buchner, Stephane Ansieau, Michael Sattler

**Affiliations:** 1 Institute of Structural Biology, Helmholtz Zentrum München, Neuherberg, Germany; 2 Biomolecular NMR and Center for Integrated Protein Science Munich at Department Chemie, Technische Universität München, Garching, Germany; 3 Institut National de la Santé Et de la Recherche Médicale U590, Centre Léon Bérard, Université Claude Bernard Lyon I, Lyon, France; 4 Tianjin Institute of Industrial Biotechnology, Chinese Academy of Sciences, Tianjin, China; 5 Dipartimento di Chimica, Università degli Studi di Napoli “Federico II”, Napoli, Italy; 6 Novartis Vaccines and Diagnostics, Siena, Italy; Spanish National Cancer Center, Spain

## Abstract

DEAF-1 is an important transcriptional regulator that is required for embryonic development and is linked to clinical depression and suicidal behavior in humans. It comprises various structural domains, including a SAND domain that mediates DNA binding and a MYND domain, a cysteine-rich module organized in a Cys_4_-Cys_2_-His-Cys (C4-C2HC) tandem zinc binding motif. DEAF-1 transcription regulation activity is mediated through interactions with cofactors such as NCoR and SMRT. Despite the important biological role of the DEAF-1 protein, little is known regarding the structure and binding properties of its MYND domain.

Here, we report the solution structure, dynamics and ligand binding of the human DEAF-1 MYND domain encompassing residues 501–544 determined by NMR spectroscopy. The structure adopts a ββα fold that exhibits tandem zinc-binding sites with a cross-brace topology, similar to the MYND domains in AML1/ETO and other proteins. We show that the DEAF-1 MYND domain binds to peptides derived from SMRT and NCoR corepressors. The binding surface mapped by NMR titrations is similar to the one previously reported for AML1/ETO. The ligand binding and molecular functions of the related BS69 MYND domain were studied based on a homology model and mutational analysis. Interestingly, the interaction between BS69 and its binding partners (viral and cellular proteins) seems to require distinct charged residues flanking the predicted MYND domain fold, suggesting a different binding mode. Our findings demonstrate that the MYND domain is a conserved zinc binding fold that plays important roles in transcriptional regulation by mediating distinct molecular interactions with viral and cellular proteins.

## Introduction

Initiation of transcription is tightly regulated by a complex interplay of transcription factors which can either directly bind to the DNA or to other transcriptional co-factors. Mutations in these proteins are linked to cancer and other pathologies. DEAF-1 is an important transcriptional regulator that is required for embryonic development [1]–[3] and linked to clinical depression and suicidal behavior in humans [4]. Michelson et al, showed that deletion of the MYND domain in human DEAF-1 results in a protein less effective than the full length protein in transcriptional repression of nuclear ribonucleoprotein A2/B1 Promoter [2]. DEAF-1 has also been identified as a protein partner of LMO4 that plays important roles in mammary gland development and breast cancers. Consistently, overexpression of DEAF-1 *in vitro* as well as *in vivo* was found to promote mammary epithelial proliferation [5]. Furthermore, DEAF-1 deficient mice displayed phenotypic abnormalities similar to those observed in LMO4 mutants including exencephaly, transformation of cervical segments, and rib cage abnormalities [6]. Through LMO4, DEAF-1 also interacts with the tumor suppressor BRCA1, potentially linking DEAF-1 to breast cancer development [2], [7], [8].

The DEAF-1 protein contains two conserved domains, the SAND (Sp100, AIRE-1, NucP41/75 and DEAF-1) domain, originally named KDWK after the conserved amino acid core, and a cysteine rich region called the MYND (MYeloid translocation protein 8, Nervy and DEAF-1) domain [1]. To the best of our knowledge, it is the only known mammalian protein that contains both domains. Transcriptional regulation by DEAF-1 involves recognition of target DNA sequences comprising TTCG [1], [3] or TTCGG [9] motifs in Drosophila and humans, respectively by the SAND domain [1], which was previously found to adopt a novel DNA binding fold [10]. The MYND domain of DEAF-1 failed to specifically bind DNA, but may still play a role in transcriptional regulation by interacting with corepressor proteins. For example, in the chimeric AML1/ETO protein produced by the 8;21 translocation associated with 12–15% of acute myeloid leukemias, the ETO MYND domain binds the SMRT and NCoR corepressors [11]–[13], and MYND domain of the adenoviral protein BS69 was shown to interact with NCoR [14]. BS69 also interacts with the adenoviral oncoprotein E1A as well as with the EBNA2 (Epstein-Barr virus-induced Nuclear Antigen2) protein. These interactions depend on the presence of a PXLXP amino acid motif (X: any amino acid). Interestingly, whereas MYND domains in the Bra-1 and Bra-2 proteins were found to bind E1A and EBNA2, no interaction of the RACK7 or ETO MYND domains with these viral proteins was observed [15], suggesting different binding specificities among MYND domains.

MYND domains comprise 40 to 60 residues with two conserved zinc-binding motifs that are often located at the amino or carboxyl termini of the corresponding full length proteins. MYND domains have two consensus zinc-binding motifs C-X-X-C and C/H-X-X-X-C with a characteristic conserved spacing of cysteine and histidine residues. Structures of the MYND domains of AML1/ETO [13] and in several paralogs of SMYD proteins have been reported recently [16]–[23] and demonstrated that the tandem zinc-binding motifs are organized in cross-brace topology similar to that observed in RING (Really Interesting New Gene-1) finger domains [24]. The structure of ETO MYND bound to a peptide motif in the SMRT co-repressor revealed molecular details for the recognition of a proline-rich PPPLI motif in SMRT and NCoR corepressors [13]. An intact zinc coordination by the SMYD3 MYND domain was found to be required for an interaction with the NCoR corepressor in coimmunoprecipitation experiments [22], while another report suggested that the SMYD3 MYND domain could be involved in DNA binding [17]. However, based on the conservation of MYND domain residues that are important for corepressor binding by ETO it seems plausible that SMYD MYND domains may also bind SMRT and NCoR corepressors. However, this has not been demonstrated experimentally.

Despite the important biological role of the DEAF-1 protein little is known regarding the structure and binding properties of its MYND domain. Here, we report the solution structure, backbone dynamics and ligand binding properties of the human DEAF-1 MYND domain encompassing residues 501–544 using NMR spectroscopy. The structure presented herein corrects our previous report [25], [26] of an incorrect structure of the DEAF-1 MYND. Compared to the previous study where a longer construct of the DEAF-1 MYND domain was used the present study is based on a shorter construct that yields significantly improved NMR data. The structure of the DEAF-1 MYND domain reveals a tandem zinc-binding fold with a cross-brace topology, similar to other MYND domains. We show that DEAF-1 MYND binds to peptides derived from SMRT and NCoR and have mapped the binding surface by using NMR titrations. In addition, we have characterized the ligand binding properties and molecular functions of the related BS69 MYND domain based on a homology model and mutational analysis. Our findings demonstrate that the conserved MYND domain fold found in different transcriptional regulators is a versatile scaffold that may support distinct molecular interactions with viral and cellular proteins.

## Results

### Zinc binding of the DEAF-1 MYND domain

**Figure 1:**
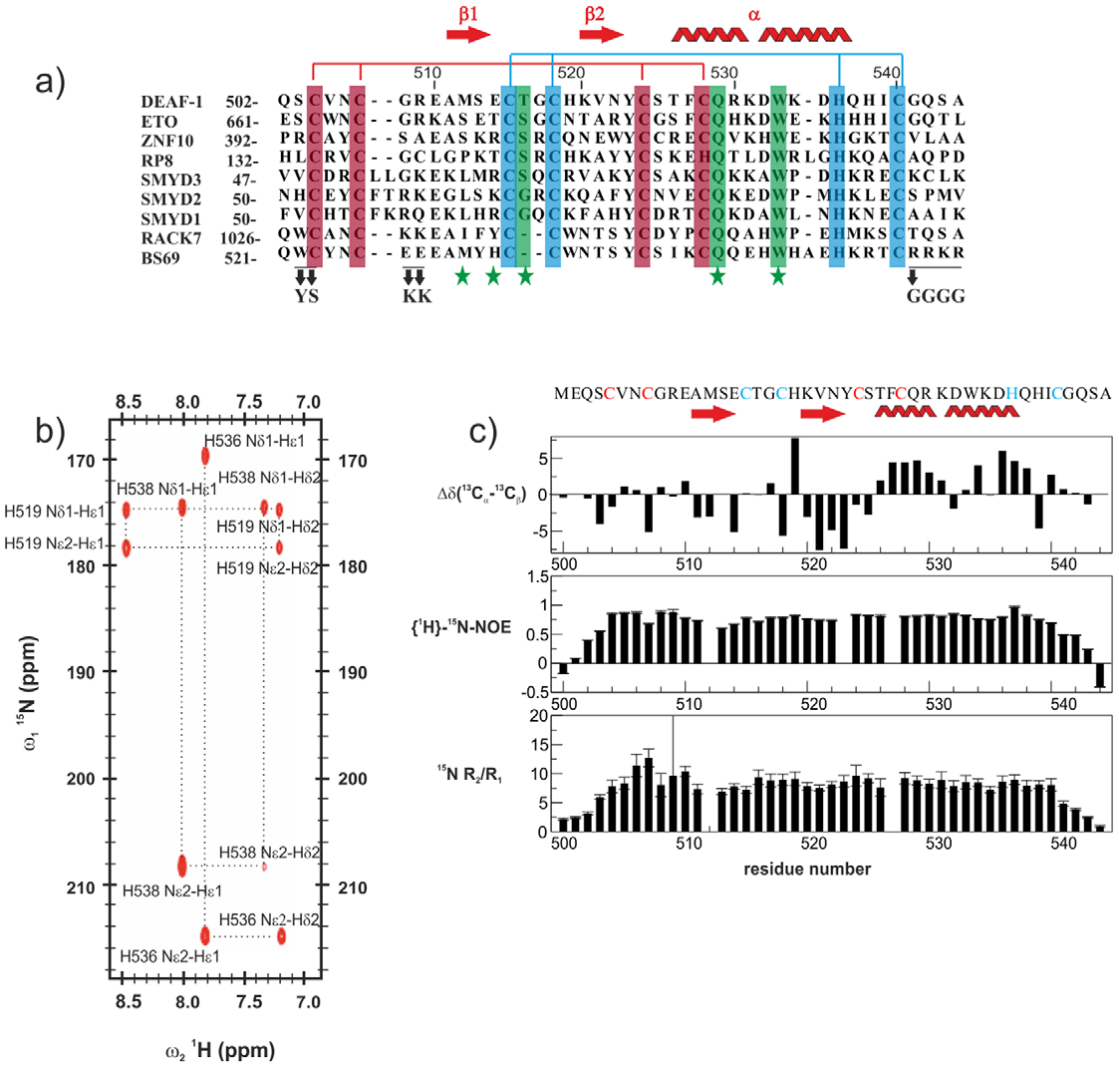
Primary sequence and NMR analysis of the DEAF-1 MYND domain. (a) Sequence alignment of different MYND domains. Residues coordinating the first and second zinc ions are highlighted with red and blue background, respectively. Residues involved in binding to corepressor peptides are indicated with a green star at the bottom, and those interacting through their side chains are highlighted in green. The positions of mutations performed on BS69 are indicated at the bottom. (b) Long range ^1^H, ^15^N HSQC spectrum correlating H^ε1^ and H^δ2^ to N^δ1^ and N^ε2^ through ^2^ *J*_HN_ and ^3^ *J*_HN_ couplings (Pelton et al 1993). The spectrum reveals a different protonation pattern for each histidine sidechain corresponding to the three possible tautomeric states. (c) ^13^C secondary chemical shifts (top), {^1^H}-^15^N heteronuclear NOE (middle), and ^15^N R_2_/R_1_ relaxation rates ratio (bottom) are plotted versus DEAF-1 MYND residue numbers. The secondary structure elements and the amino acid sequence of the protein are indicated at the top of the figure. Residues coordinating the first and second zinc are colored red and blue respectively.

For structural and biochemical studies various constructs of the DEAF-1 MYND domain (Figure 1a) were cloned and expressed in *E. coli*. A protein comprising residues 501–544 of human DEAF-1 representing the globular fold of the MYND domain was used for further studies. The tandem zinc fingers of MYND domains bind two zinc ions in a tetrahedral coordination via seven cysteine and one histidine side chain. The dependence of the MYND domain fold on zinc coordination was confirmed by the addition of 5 mM EDTA to the DEAF-1 MYND domain. The addition of this metal cation chelator results in a poorly dispersed NMR spectrum, particularly in the ^1^H chemical shift region around 6–9 ppm, characteristic of an unfolded protein (Figure S1a). This confirms the importance of zinc ions in maintaining the fold, as has been reported for other MYND domains [13].The DEAF-1 MYND domain contains seven cysteines that are all involved in zinc chelation and are identified based on the sequence conservation (Figure 1a). We used NMR to identify which of the three histidines is involved in zinc coordination and characterized their tautomeric states. Different patterns were observed in a long-range ^1^H,^15^N HSQC experiment correlating histidine H^ε1^ and H^δ2^ protons to their neighboring nitrogen N^δ1^ and N^ε2^ atoms [27]. Figure 1b shows that N^δ1^ and N^ε2^ of His519 have very similar chemical shifts, indicating a doubly protonated state, where the charge is distributed between the two nitrogen atoms. In contrast the imidazole rings of histidines H536 and H538 are protonated at their N^δ1^ and N^ε2^ nitrogens, respectively. Binding to a zinc ion usually involves N^ε2^, but coordination via N^δ1^ has also been observed in some RING fingers [24], [28]. On the basis of the tautomeric states we can thus not exclude that H538 might mediate the zinc coordination. However, mutation of His538 to Ser results in a well dispersed spectrum that strongly resembles the wild type one (Figure S1b) indicating that H538 is not required for maintaining the fold of the DEAF-1 MYND domain and thus is not involved in zinc coordination. Taken together, these experiments demonstrate that zinc coordination of the DEAF-1 MYND domain involves His536 and the seven cysteine residues.

### Solution structure of the DEAF-1 MYND domain

**Figure 2:**
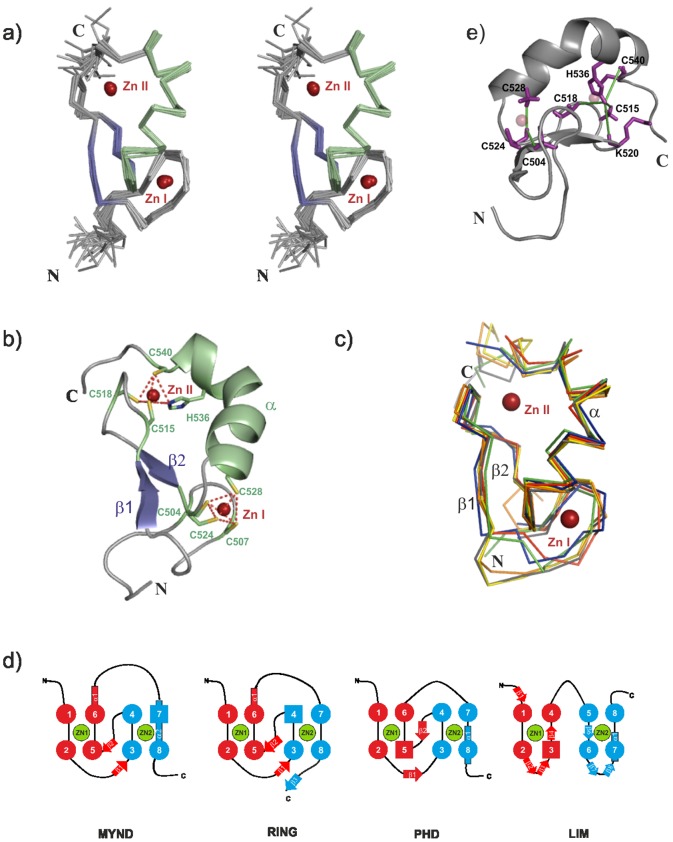
Three-dimensional structure of the DEAF-1 MYND domain. (a) Stereo view of the ensemble of the twenty lowest energy structures of the DEAF-1 MYND domain. α helices and β strands are colored in green and purple respectively, whereas zinc atoms are depicted as red spheres. (b) Ribbon representation of the DEAF-1 MYND domain. Side-chains of residues coordinating the zinc atoms are shown as sticks. The zinc coordination geometry is indicated by red dotted lines. (c) Superposition of DEAF-1 (green), ETO (red), ZNF10 (Blue), SMYD1 (yellow), SMYD2 (orange) and SMYD3 (gray) MYND structures shown in ribbon representation. The two zinc ions are depicted as red spheres. (d) Schematic representation of the zinc-binding pattern and secondary structure elements in MYND, RING, PHD and LIM domains. (e) Cartoon representation of DEAF1-MYND domain. Side chains of residues for which medium and long-range NOEs are observed that unambiguously define the cross-brace zinc binding topology are shown in magenta. Green lines indicate NOEs between C524 H^N^/C504 H^β*^, C524 H^N^/C528 H^β*^ for the first binding site; and H536 H^ε1^/C540 H^N^, H536 H^ε1^/C518 H^β1^, H536 H^ε1^/K520 H^N^, and H536 H^ε1^/C515 H^β2^ for the second binding site.

The solution structure of the MYND domain was determined using standard triple resonance NMR experiments [29]. A summary of NMR data and the secondary structure is shown in Figure 1c. Distance and orientational restraints were derived from NOE intensities and residual dipolar couplings, respectively. In addition, backbone torsion angle restraints were defined based on secondary chemical shifts using TALOS+ [30]. Special care was taken to ensure the tetrahedral zinc coordination geometry during the structure calculations by a combination of distance and angle constraints as described in the materials and method section. Structures were calculated using CYANA [31] and further refined using ARIA/CNS [32], [33]. Initial calculations in CYANA using only NOE-derived distance restraints and dihedral angle restraints identified the protein fold with the candidate residues for zinc coordination being in close proximity, and consistent with a cross-brace arrangement of the two zinc-binding motifs. Once the topology of the Zn^2+^-coordinated residues was confirmed, subsequent CYANA structure calculations employed distance restraints that imposed tetrahedral Zn^2+^-coordination to Cys and His residues. The zinc coordination has been confirmed by the unambiguous assignment of medium and long-range NOEs, for example between C524 H^N^ and C504 H^β*^, C524 H^N^ and C528 H^β*^ for the first binding site; and between H536 H^ε1^ and C540 H^N^, H536 H^ε1^ and C518 H^β1^, H536 H^ε1^ and K520 H^N^, H536 H^ε1^ and C515 H^β2^ for the second binding site (Figure 2e). The final CYANA structures were refined in a box of explicit water molecules using ARIA/CNS [34], adapted to ensure tetrahedral zinc coordination geometry during this final refinement. The statistics of structure determination and quality analysis are reported in Table 1. The final ensemble of twenty structures for the DEAF-1 MYND domain is shown in Figure 2. Consistent with ^13^C secondary chemical shifts (Figure 1c) few regions with secondary structure are observed. The structure of the MYND domain is well-defined by characteristic NOEs. It comprises a short β hairpin and an α-helix with a kink around Asp532, which is also consistent with ^13^C secondary chemical shifts.

**Table 1:** Structural statistics of the DEAF-1 MYND domain.

**NOE-based distance restraints** 1	
short-range, |i-j|< = 1	439
medium-range, 1<|i-j|<5	180
long-range, |i-j|> = 5	183
Total	802
**Dihedral angle restraints** 2	
Φ+Ψ angles	62
**Residual dipolar coupling restraints** 3	
H^N^N+NC′+H^N^C′+H^α^ C^α^	63
**Coordinate RMSD for residues 502–541(Å)**	
Backbone	0.36±0.10
Heavy atoms	1.10±0.18
**Consistency**	
RDC Q-factor	0.09±0.002
**Ramachandran plot statistics (%)** 4	
Most favoured regions	80.3
Allowed regions	18.9
Generously allowed regions	0.8
Disallowed regions	0.0

Statistics are given for the 20 lowest energy structures after water refinement out of 100 calculated.

1 Distance restraints were derived from NOE peak intensities using CYANA [31], and then introduced as unambiguous distances in CNS. No distance restraint was violated by more than 0.5 Å.

2 Torsion angles were predicted using TALOS+ [30]. No dihedral angle restraint was violated by more than 5°.

3 RDC restraints were incorporated using a harmonic potential. Force constants of 0.2, 0.1, 0.3, and 0.6 kcal mol^−1^ Hz^−2^ for H^H^N, NC′, H^N^C′ and H^α^ C^α^ respectively, were used to reflect the estimated error in the measurement.

4 Ramachandran plot statistics were obtained using PROCHECK [64] for residues 502–541.

The structure of the DEAF-1 MYND domain presents a cross-brace zinc-binding topology (Figure 2), as observed for other MYND domains [13], [16], [19], [20]. The ββα secondary structure and cross-brace zinc-binding topology is shared with RING finger domains, and is common to many extracellular small domains stabilized by disulphide bonds [35]. The first two zinc coordinating cysteine residues in each binding site are located in loop regions, while the last two coordinating residues are within and flanking the α-helix. In the final structures of the DEAF-1 MYND domain the zinc ions show a perfect tetrahedral coordination (Figure 2b). The two zinc atoms are 14 Å far apart, which is another common characteristic of MYND and RING finger domains reflecting the highly conserved spacing between the zinc chelating residues (Figure 1a). The structure of DEAF-1 MYND superimposes very well with other MYND domains, such as those of ETO, ZNF10, SMYD1, SMYD2 and SMYD3 with coordinate root-mean-square deviations for the backbone atoms of 0.99, 1.19, 1.05, 1.07 and 0.99 Å, respectively (Figure 2c). The cross-brace zinc-binding topology of the MYND domain and the arrangement of secondary structure elements strongly resemble RING and PHD finger domains. On the contrary, LIM domains show a sequential zinc-binding topology resulting in two independent zinc-binding sites (Figure 2d).

### Backbone dynamics of the DEAF-1 MYND domain

NMR ^15^N relaxation measurements (^15^N R_1_ and R_2_ and {^1^H}-^15^N heteronuclear NOE; Figure 1c) were analyzed using a model-free approach [36], [37] as implemented in TENSOR2 [38]. The analysis indicates a tumbling correlation time τ_c_ of 6.1 ns for the DEAF-1 MYND domain at 22°C. This value is significantly larger than expected for a protein of this size, suggesting possible oligomerization of the MYND domain in solution, as has been reported for other MYND domains [13]. Oligomerization is however thought not to be biologically relevant but rather is associated with the high concentration used for the NMR experiments. Analytical ultracentrifugation (AUC) data indicate that the dimerization dissociation constant is 0.5±0.1 µM (Fig. S2a). We identified the residues involved in the oligomerization based on NMR relaxation data and chemical shift changes observed in NMR spectra recorded at different sample concentrations (Fig. S2c). Amide NMR signals of residues located in the vicinity of the first zinc-binding site (Asn506, Cys507, Arg509, Glu510) exhibit slightly increased R_2_/R_1_ ratios (Figure 1c, middle) compared to the values for other residues in the structured region of the MYND domain. This suggests the presence of motions in the micro- to milliseconds time scale range, characteristic of exchange phenomena between different conformations, such as monomeric and dimeric states of the protein. Comparison of ^1^H,^15^N HSQC spectra acquired at different concentrations show line-broadening of certain peaks even at concentrations as low as 10 µM with some resonances broadened beyond detection (Fig. S2b), consistent with the dimerization constant obtained from analytical ultracentrifugation data (Fig. S2a). ^1^H,^15^N HSQC experiments recorded at different salt concentrations (Fig. S3a) and pH (Fig. S3b), as well as ^15^N relaxation rates (Fig. S3c) show that these buffer conditions do not affect the oligomerization. Most of the residues that exhibit the concentration-dependent line-broadening are located in the first zinc-binding site and thereby identify the dimerization surface. Given that this dimerization interface is far away from the binding site of the MYND domain with co-repressors (see below; Fig. S2c) it can be safely excluded that the dimerization observed *in vitro* could interfere with ligand binding by the MYND domain (see Supplementary Information; Figs. S2, S3 for further details).

### Protein-protein interactions mediated by the DEAF-1 MYND

**Figure 3:**
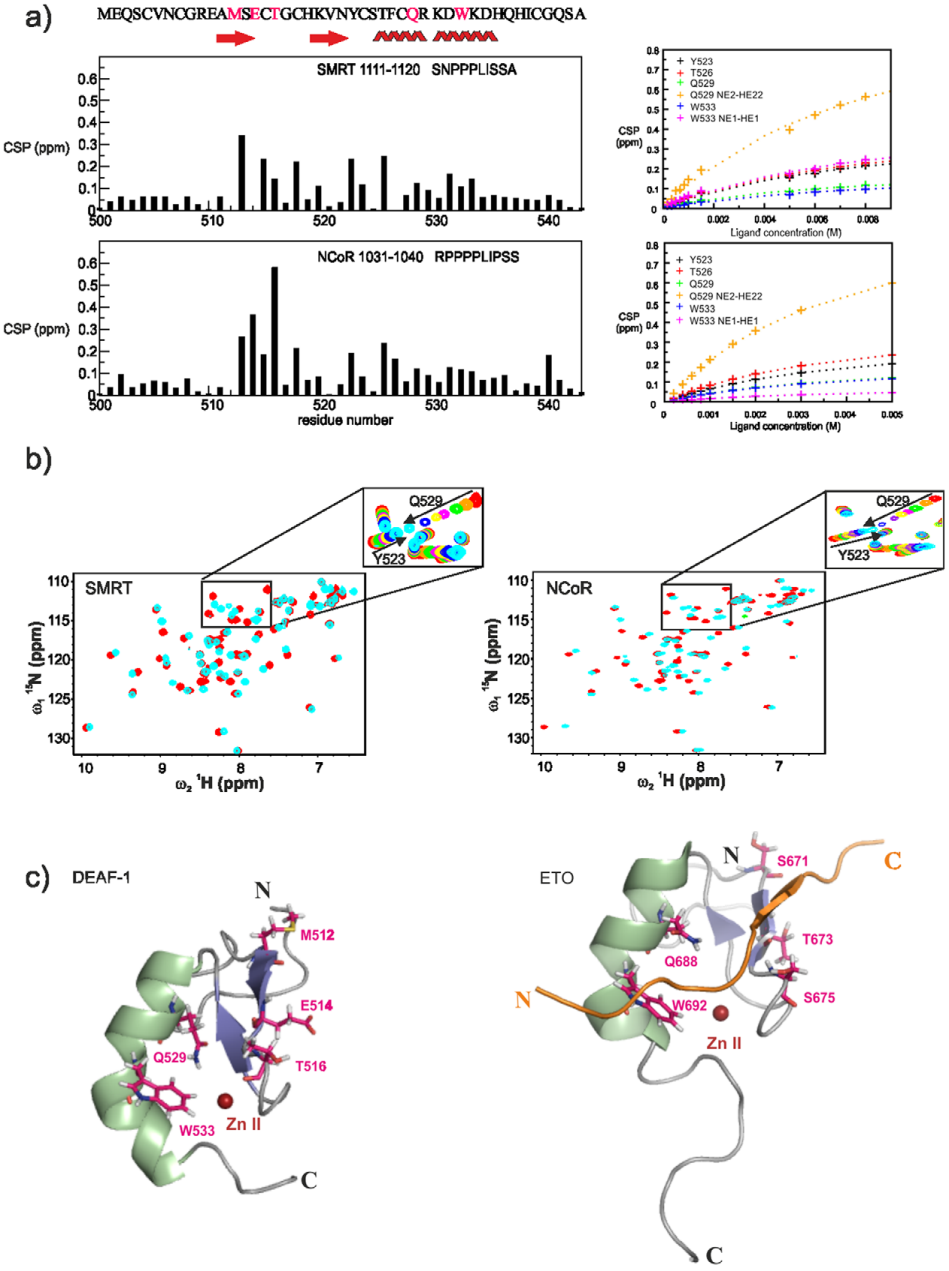
Binding of DEAF-1 MYND to SMRT and NCoR peptides. (a) Chemical Shift Perturbations (CSP, see methods) observed for the interaction between DEAF-1 MYND domain and SMRT (top) and NCoR (bottom) corepressor peptides. The sequence of SMRT and NCoR peptides used for the titrations are indicated in each graph. Secondary structure elements and amino acid sequence of the DEAF-1 MYND domain are shown at the top of the panel. Residues expected to interact with the corepressors are colored magenta. CSPs of residues that are most strongly affected are shown (cross symbols) as a function of ligand concentration for both titration with SMRT (top right) and NCoR (bottom right). The observed CSPs were fitted to a binding isotherm yielding dissociation constanst of 5.30±0.54 mM and 3.08±0.12 mM for the SMRT and NCoR peptides, respectively. The binding curves are shown as dashed lines. (b) Superposition of ^1^H, ^15^N HSQC spectra of a 1 mM sample of the free DEAF-1 MYND domain (red) and upon addition of unlabeled corepressor peptides (cyan) up to a final concentration of 8 mM and 5 mM of SMRT and NCoR peptide (1∶8 and 1∶5 molar ratio), respectively. The intermediate steps of each titration are zoomed for a sub-region of the corresponding spectrum. In either case binding takes place on the fast exchange regime with respect to the chemical shift time scale. (c) Ribbon representation of DEAF-1 (left) and ETO (right) MYND domains. Residues experiencing the largest chemical shift perturbation upon addition of the corepressor peptides are shown as magenta sticks. In the ETO-SMRT complex structure the corresponding residues are shown as sticks as well, and the SMRT ligand peptide is colored orange and shown in cartoon representation.

Although the DEAF-1 MYND domain adopts a ββα fold common to many DNA binding zinc finger proteins, it failed to interact specifically with DNA [1]. Rather DNA binding of DEAF-1 occurs through its SAND domain [1]. In contrast, MYND domains are thought to mediate protein-protein interaction [1], [39] linked to the recruitment of corepressors [3]. In fact, this has been reported for the ETO MYND domain, which binds the SMRT and NCoR corepressors [12], [13], [40] through a common PPPLI motif [13]. The high degree of sequence and structural homology between ETO and DEAF-1 MYND domains suggests that the DEAF-1 MYND domain might also interact with SMRT and NCoR. We noted that especially residues important for corepressor binding in ETO are conserved in DEAF-1 (Met512, Glu514, Thr516, Gln529, Trp533; Figure 1a). We therefore used NMR titrations to test whether the DEAF-1 MYND domain does bind peptides derived from SMRT and NCoR. Given the conservation of MYND domain residues we expected that the interaction of the DEAF-1 MYND domain would rely on similar peptide sequences that were reported to bind to ETO [13]. NMR titrations with a 1 mM sample of DEAF-1 MYND and peptides encompassing residues 1111–1120 of SMRT or 1031–1040 of NCoR (Figure 3a) indicate a specific interaction. Binding occurs in the fast-exchange regime on the NMR chemical shift time scale, consistent with a low binding affinity (Figure 3). Notably, DEAF-1 MYND residues that experience large chemical shift changes during the titration (Figure 3a and 3b) are those expected to interact based on the sequence comparison with ETO (see above) or residues located in their vicinity (Figure 3c). The chemical shift perturbations (CSP) observed during titration experiments were fitted to a binding isotherm yielding dissociation constants (K_D_) of 5.30±0.54 mM and 3.08±0.12 mM for SMRT and NCoR peptides, respectively. NMR ^15^N relaxation rates measured in the absence and presence of a large excess of peptide show no significant changes in dynamic properties (data not shown). This confirms that ligand binding does not affect the oligomerization of the domain as expected (see above).

### Functional characterization of the BS69 MYND domain

**Figure 4:**
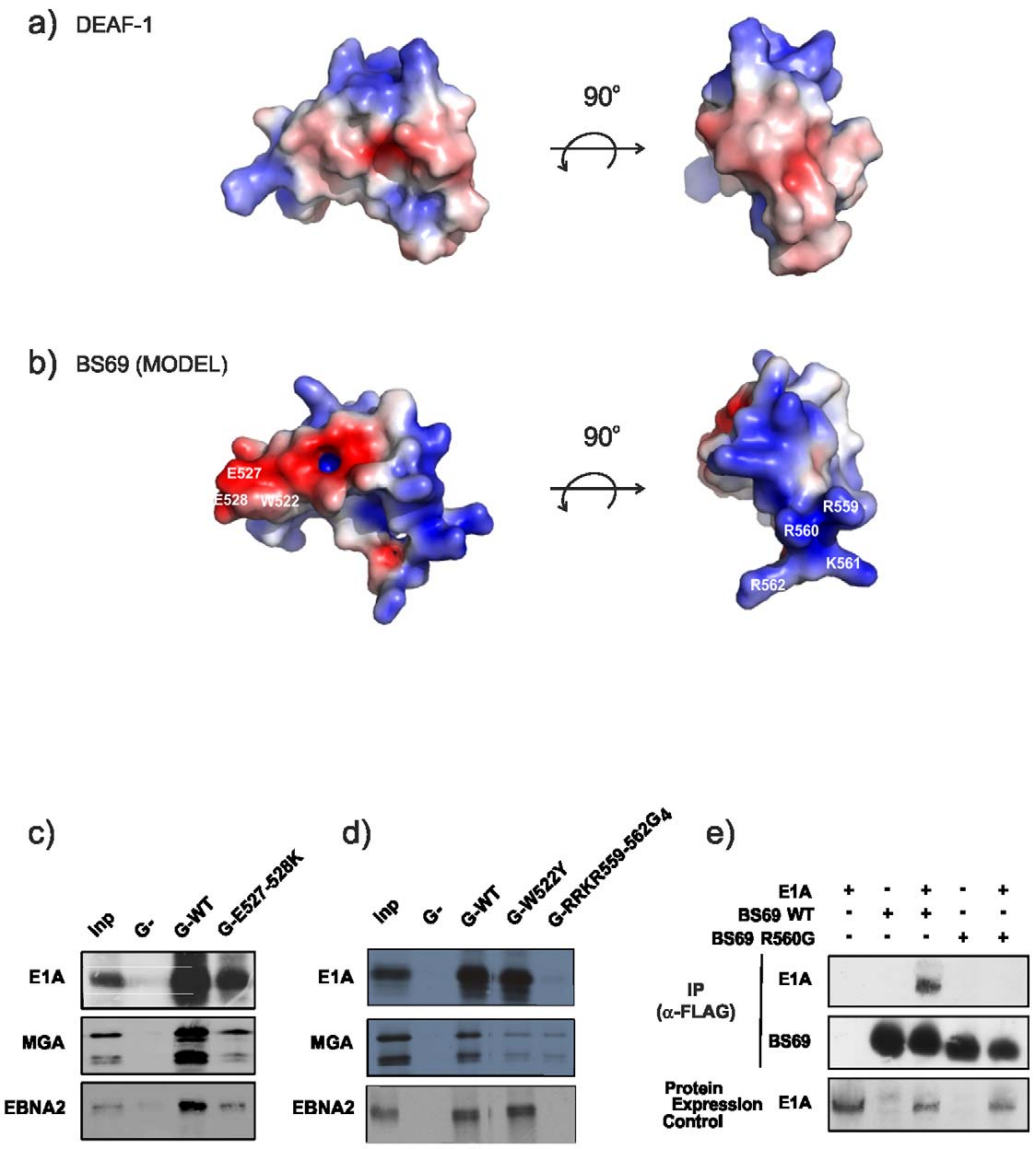
Binding studies and mutational analysis of BS69 MYND domain. (a) Two views of an electrostatic surface representation of the DEAF-1 MYND domain. Positive (blue) and negative (red) electrostatic surface potential is shown at ±3 k_B_ T/e^−^ and was determined using the program APBS [66] in Pymol (www.pymol.org). (b) Corresponding surface views for the BS69 MYND structure obtained from homology modelling. Positive (blue) and negative (red) electrostatic surface potential is displayed at ±3 k_B_ T/e^−^. The location of the residues that were mutated for binding studies are labelled on the surface representation of the BS69 homology model. (c),(d) Analysis of binding of E1A, EBNA2 and MGA to wild-type or mutant BS69 proteins expressed as a GST-fusion protein or to the GST (G−) moiety as control; Inp: Input 10%; G-RRKR-559-562G4: mutation of residues 559–562 into four glycines. (e) A single point mutation in BS69 abrogates the binding to E1A. QT6 fibroblasts were transfected with 12SE1A and/or FLAG-tagged BS69 expression vectors as indicated. Cellular complexes were immunoprecipitated with an M2 anti-FLAG antibody. Co-immunoprecipitated and ectopic expressions of E1A were revealed by immunoblotting using an M73-E1A antibody.

In order to investigate whether the structural characteristics and ligand interactions of MYND domains are conserved we studied the BS69 MYND domain. This domain was previously shown to mediate binding to the NCoR corepressor [14], but also found to interact with cellular partners, such as the Myc-related protein MGA and the viral proteins E1A and EBNA2 depending on the presence of a PXLXP sequence motif in these binding partners [41]. In spite of considerable attempts we were not able to prepare recombinant BS69 MYND in *E. coli* for structural and biochemical studies. However, based on the sequence conservation (∼40% identity) between the two domains, we calculated a homology model for the BS69 MYND domain based on the DEAF-1 structure using MODELLER [42]–[45]. Interestingly, the BS69 MYND model reveals a highly charged surface with a positive face consisting of C-terminal residues on one side, and a negatively charged region on the other side (Figure 4b). The binding of the BS69 MYND domain to MGA, E1A and EBNA2 has been studied by mutational analysis using *in vitro* translated GST-tagged BS69 protein, encompassing the MYND domain. As expected, mutation in one of the zinc coordination sites, completely abolishes the binding to any partners [15], confirming that a structurally intact MYND domain is required for the interaction. Additional mutational analysis was performed using GST pull-down assays. Notably, a charge reversal of Glu527–Glu528 (EE527–528KK), involving residues, which are located in a region flanking the first zinc-binding module, strongly reduced the interaction with all three PXLXP ligands (Figure 4c), while the W522Y mutation mainly affects the interaction with the cellular ligand (MGA) but not the viral binding partners (Figure 4d). The BS69 MYND domain contains a set of positively charged residues located at its C-terminus that are absent from other MYND domains, in particular from RACK7, ETO and DEAF-1, which fail to interact with BS69 PXLXP ligands [46]. Mutation of the positively charged residues (RRKR559–562GGGG) abolishes binding to PXLXP ligands (Figure 4d). Individual mutations of these residues further revealed that among these four residues only Arg560 is essential for binding of BS69 to its ligands (Figure 4e; Fig. S5). Taken together the mutational analyses suggest that a positively charged face of the BS69 MYND domain, at the C-terminal end of the zinc-binding fold, is crucial for the interaction with PXLXP ligands and appears to be a unique property of the BS69 protein.

## Discussion

The solution structure of the DEAF-1 MYND domain reported here reveals a tandem C4-C2HC zinc-binding motif with a cross-brace topology. This structure corrects our previous report that was based on a C-terminally extended, longer construct of DEAF-1 MYND domain (residues 498–565), for which only few long range NOEs could be obtained (Fig. S4a,b) [25]. For this longer construct key long-range NOEs that unambiguously define the cross-brace zinc topology were only obtained with a 800 MHz NOESY experiment ([26] and Fig. S4a). The structure of the DEAF-1 MYND domain is organized in a ββα fold, which is likely to stabilize the MYND domain fold in the reducing intracellular compartment where disulfide bonds are unstable. The zinc binding topology is somewhat reminiscent of the 1–3, 2–4 disulfide bonds pattern found in extracellular domains, for example in Epidermal Growth Factor (EGF) [47]. Both the zinc coordination and the distance between the two zinc ions are shared by DEAF-1 with other MYND domains as expected from the high sequence similarity observed among this protein family. Note, that an optimized protein construct has been used for structural analysis, which removes any unstructured tails and merely encompasses the structured tandem zinc finger region of the MYND domain. This construct therefore yields high quality NMR spectra and the structural analysis presented herein corrects a previous report [25], [26] that had been conducted on a longer construct (see Supplementary Information).

Our NMR titrations using peptides derived from the silencing mediator of retinoid and thyroid receptor (SMRT) and from the nuclear receptor corepressor (NCoR), show that the binding site of DEAF-1 MYND for these corepressors resembles that of the ETO MYND domain. The interaction of these ligands to DEAF-1 MYND is about an order of magnitude weaker than the one previously reported for the ETO MYND domain [13]. This suggests that additional interactions between the full-length DEAF-1 and corepressor proteins may exist that further enhance the interaction. Nevertheless, the striking similarity of the chemical shift perturbations seen for the DEAF-1 and ETO MYND domains and the conservation of residues that are important for the interaction in ETO MYND (Figure 1a) indicates a very similar interaction of the corepressors with the DEAF-1 MYND domain. The recognition of the corepressor peptides by the ETO MYND domain involves hydrogen bonds with backbone or side chains atoms of residues that are also conserved in DEAF-1, including Gln529 [13] (Figure 1a). Another important residue for this interaction is a tryptophan (W533 in DEAF-1; Figure 1a) which packs against a proline in the SMRT ligand peptide bound to the ETO MYND domain [13]. The high degree of similarity between DEAF-1 and ETO MYND domains suggests a similar binding mode, as confirmed by the binding site mapped by our NMR chemical shift perturbations (Figure 3c).

Recently, Foreman and coworkers determined the crystal structure of a multi-domain construct of SMYD3 and found that an intact MYND domain fold is required for interaction with the NCoR corepressor [22]. Although the binding interface with NCoR has not been mapped in this study, the MYND domain residues that are important for corepressor binding are conserved in SMYD3, suggesting a similar binding mode.

Conservation of critical residues involved in the interaction may thus explain why an interaction with corepressors is widely observed among the MYND domain family. While binding to corepressors such as SMRT and NCoR seems to be a common feature of many MYND domains, including those of ETO, BS69, and BOP [12]–[14], [40], [48], [49], distinct binding specificities exist for other MYND domains, in particular for the interaction with viral proteins such as E1A and EBNA2. The interaction between BS69 and binding partners that comprise a PXLXP sequence motif [41] seems to critically depend on electrostatic contacts. A comparison of the electrostatic surface potentials of the DEAF-1 and BS69 MYND domains (Figure 4a,b, respectively) shows that DEAF-1 MYND is less charged than BS69. This is also indicated by the theoretical pI values of 8.5 and 6.8 for BS69 and DEAF-1 MYND domains respectively, obtained using ExPASy [50]. The less pronounced charge of the DEAF-1 (also seen for the ETO and RACK7 MYND domains, pI's of 6.4 and 6.9 respectively, Figure 1a) seems to correlate with a lack of binding to E1A and EBNA2. In contrast, the interaction of BS69 with these PXLXP-containing proteins depends on the presence of charged residues in the sequence. Since charge reversal of two glutamate residues (EE527–528KK) has a smaller effect, it appears that positively charged arginine and lysine residues flanking the C-terminal end of the zinc-binding fold of the BS69 MYND domain are crucial for the interaction. The positive charges of these side chains could mediate long-range electrostatic interactions with negative charges which may be located in regions flanking the PXLXP motif in the binding partners. The BS69 MYND domain interaction might thus involve a larger binding epitope of which PXLXP merely represents a conserved core motif. Additionally, aromatic residues might contribute to specific PXLXP interactions, by packing against the pyrrolidine ring of the Pro side chains. For example, a tyrosine (Y523) located between the first two zinc-coordinating Cys residues in the BS69 MYND domain only, suggests that hydrophobic interactions of this aromatic residue could further contribute to ligand binding of BS69. This might also explain why ETO MYND domain failed to interact with this PXLXP motif [15].

Although BS69 has been shown to bind to NCoR [14], which also contains a PXLXP recognition motif, the analysis of its sequence reveals that the key residues that are important for corepressor binding (i.e. T516 in DEAF-1, S673 in ETO) are not conserved. This suggests that the binding mode may differ among the MYND domain family. Supporting this hypothesis, the analysis of the BS69 (and RACK7) primary sequence shows that it lacks a Thr/Ser residue and that the two cysteines involved in the second zinc-binding site are directly consecutive without any spacing residues in between (Figure 1a). This likely affects the conformation of this region in the BS69 and RACK7 MYND domains. Additional electrostatic interactions involving residues flanking the MYND fold (Figure 4) could thus additionally contribute to ligand binding by BS69. The NCoR interaction of BS69 may depend critically on electrostatic contacts as observed for binding to MGA and E1A. In the case of SMYD1, which also lacks the Thr/Ser residue at this position, the binding to a PXLXP motif in the transcriptional activator skNAC depends on additional interactions of amino-terminal residues located in the S-sequence domain [49]. Interestingly, the SMYD1 MYND domain is highly positively charged [19], suggesting that skNAC binding may be enhanced by electrostatics interactions.

Collectively, these data suggest that variations in amino acid sequence drive distinct binding specificities of MYND domains with their cellular and viral partners, even though binding to corepressors such as NCoR and SMRT seems to be a feature that is generally shared by many MYND domains. The relatively weak interaction of DEAF-1 MYND with the corepressor-derived peptides, may be further enhanced by additional protein-protein contacts involving different regions of the full-length DEAF-1 and corepressor proteins. Thus future functional studies of the role of DEAF-1 should consider the possibility that additional domains may further enhance the interaction. The DEAF-1 and ETO MYND domains appear to use the same binding mode when interacting with corepressors, and both fail to bind to MGA, E1A and EBNA2 proteins. In contrast, MYND domains that are more distantly related, such as BS69, may favor electrostatic interactions through their specific charged residues. Future structure/function studies will further highlight the importance of MYND-mediated protein-protein interactions in transcriptional regulation with viral and cellular proteins.

## Materials and Methods

### Cloning, protein expression and sample preparation

The DNA fragments comprising the human DEAF-1 MYND domain (residues 501–544 and 496–565 for the short and long constructs respectively) plus an N-terminal insertion of three amino acids GAM, were PCR-amplified from Human cDNA. The forward primer: AAACCATGGAGCAGTCCTGCGTTAAC containing *Nco*I site and the reverse primer: AAAGGTAGGTCATGCTGACTGGCCGCATATG containing *Kpn*I. site were designed to clone MYND domain into the expression vector pETMThx, which was modified from pET24d to include an N-terminal poly-histidine tag, thioredoxin as a fused protein and a TEV cleavage site.

The DEAF-1 MYND constructs were transformed into *E.coli* strain BL21(DE3)(Novagen, USA). To prepare ^15^N, ^13^C and/or ^15^N isotope-labeled proteins expression was carried out in M9 medium containing [U-^13^C] glucose and/or [U-^15^N] NH_4_Cl as sole carbon and nitrogen sources. Overnight cultures were grown in LB medium at 37°C, diluted 50 fold into the M9 medium, continuously grown at 37°C to an OD_600 nm_ of 0.6. The culture was induced with 1 mM IPTG. During the induction 0.3 mM ZnCl_2_ was added and the culture was switched to 20°C for 10–16 hours. All media contained 50 µg/ml kanamycin.

Bacteria cells were harvested by centrifugation at 8,000 g for 30 min and re-suspended in 3 ml/g (wet weight) lysis buffer (50 mM Tris-HCl, pH 8.0, 300 mM NaCl, 5 mM imidazole), supplemented with protease inhibition cocktail complete (Roche, Germany) and DNase I (10 µg/ml). Cells were lysed by pulsed sonification (6 min, 30% power, large probe, Fisher Scientific model 550). Cell debris was removed by centrifugation at 15,000 g for one hour. All proteins were purified by two steps of chromatography. The supernatants were applied to Ni^2+^-NTA (QIAGEN, Germany), equilibrated with the lysis buffer. The bound proteins were eluted with 400 mM imidazole in the lysis buffer. The N-terminal His-tag and the fusion thioredoxin were removed by adding 1∶50 (molar ratio) TEV protease, supplemented with 2 mM DTT to the pooled protein solution. After removing the residual DTT, using a desalting column, PD10 (Amersham Biosciences, Sweden), the TEV protease and cleaved His-tag were removed by an additional Ni-chelating affinity chromatography.

The flow through from the second Ni-chelating affinity chromatography was collected, concentrated and loaded on to a gel filtration HiLoad Superdex 75 16/60 column (Amersham Biosciences, Sweden) previously equilibrated with 50 mM phosphate buffer, pH 6.5, containing 100 mM NaCl, 2 mM DTT. The fractions containing MYND proteins were pooled and concentrated to 0.3–1 mM for NMR measurements.

### NMR spectroscopy

The chemical shifts of the DEAF-1 MYND domain were assigned using standard heteronuclear experiments acquired at 295 K on a 1 mM uniformly ^15^N/^13^C labeled sample in 90% H_2_O/10% D_2_0 [29]. Experiments were carried out on Bruker spectrometers operating at a proton frequency between 500 and 900 MHz. All spectra were processed using the package NMRPipe/NMRDraw [51]. For structure determination, a 2D NOESY was recorded on a 600 MHz spectrometer equipped with a TCI cryo-probehead, whereas ^15^N- and ^13^C-edited NOESY spectra were recorded on an Avance 900 spectrometer equipped with a TXI cryo-probehead with a mixing time of 80 ms (100 ms for the aromatic ^13^C-edited NOESY spectrum). Data were analyzed using Sparky [52].

A 50 mg/ml phage solution was used to weakly align the sample in an anisotropic phase, resulting in a ^2^H quadrupolar splitting of ca 20 Hz. H^α^-C^α^, H^N^-C′, N-C′ and H^N^-N residual dipolar couplings (RDCs) were recorded on a 600 MHz spectrometer equipped with a TCI cryoprobehead using experiments described elsewhere [53]–[55].

NMR relaxation measurements including ^15^N longitudinal (R_1_), transverse (R_2_) relaxation rates, and the {^1^H}-^15^N heteronuclear Overhauser (NOE) effect were carried out on the in-house 750 MHz spectrometer equipped with a TXI probehead using standard methods [56]. For R_1_ and R_2_ relaxation rates, ten different time-points were recorded in an interleaved manner. Peak intensities were fitted to a two-parameter exponential decay using NMRViewJ [57].

Dissociation constants for the interaction of DEAF-1 MYND with proline-rich peptides derived from SMRT and NCoR corepressors were determined from fitting chemical shift changes monitored in NMR titration experiments to a binding isotherm. A 1 mM solution of ^15^N-labeled MYND domain was titrated with peptides SNPPPLISSA (SMRT) and RPPPPLIPSS (NCoR) up to a 9-fold and 5-fold molar excess, respectively. The changes in ^1^H and ^15^N chemical shifts are referred to as chemical shift perturbations (CSP) and were calculated as:
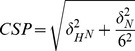



The CSPs were fitted to a binding isotherm using the equation:
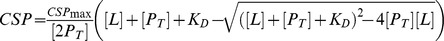
where CSP, is the chemical shift perturbation at a given peptide concentration [L], CSP_max_ is the chemical shift perturbation at saturation, [*P_T_*] is the total protein concentration, and *K_D_*, the dissociation constant.

### Structure calculation

First structure calculations were performed with CYANA [31] using NOE cross-peaks that were automatically assigned and manually checked, combined with distance restraints that impose tetrahedral Zn^2+^-coordination, torsion angle restraints derived from chemical shifts using TALOS+ [30] and RDC restraints. For the latter, the axial component and rhombicity of the alignment tensor were determined by CYANA 3.0 using a grid search approach, the result being in full agreement with what was obtained from the analysis of the RDC distribution [58]. Distance restraints derived from the CYANA calculations, together with torsion angle and RDC restraints were used in Aria1.2 for a water refinement calculation [59]–[63]. The zinc coordination geometry was defined and maintained by distance (Zn-S^γ^ 2.3 Å and Zn-N^ε2^ 2.0 Å) and angle (S^γ^-Zn-S^γ^, N^ε2^-Zn-S^γ^, Zn-S^γ^-C^β^ 109.5° and Zn-N^ε2^-C^δ2^ 120°) restraints. This procedure ensures a proper tetrahedral zinc coordination, which is not fulfilled in most of the NMR-derived zinc-binding folds found in the Protein Data Bank. In this final step a total of 100 structures were calculated from which the twenty lowest energy structures were used for quality and structure validation using the iCING web interface (http://nmr.cmbi.ru.nl/icing/) and PROCHECK [64]. The structure of the BS69 MYND domain was generated by comparative modeling with the program MODELLER [42]–[45] based on sequence alignment using the coordinates of DEAF-1 MYND domain as template. Molecular images were generated using PYMOL (www.pymol.org).

### BS69 binding experiments

Mutations were performed using the QuickChange mutagenesis kit (Stratagene). Wild type and mutants BS69 GST- fusion proteins encompass the 150 last C-terminal residues of the protein (BS69Δ431) [65]. GST-protein production, GST pull-down assay, cell transfections and immunoprecipitation experiments have been previously described [41]. For immunoprecipitation experiments, briefly, the BS69 fragment (aa 451–562 R560G) was subcloned as the FLAG-pcDNA3 vector. Combination of BS69 and 12SE1A expression vectors were transiently transfected in quail fibroblasts (QT6). Cells were harvested 24 h post-transfection, lysed and successively incubated with an anti-FLAG M2 antibody (Integra Biosciences) and Protein A sepharose (Amersham Biosciences Inc.). After separation of proteins on SDS-PAGE, BS69 and E1A were visualized with an M2 anti-FLAG antibody and an M73 anti-E1A antibody (Calbiochem) respectively.

## Supporting Information

Figure S1 **Dependence of the MYND domain fold on zinc coordination.** a. Overlay of 1H 1D spectra obtained from the wild type protein in the absence (red) and presence (cyan) of EDTA. b. Overlay of 1H-15N HSQCs of the wild type protein (red) and H538S mutant (cyan).
(TIF)

Figure S2 **Oligomerization of DEAF-1 MYND domain.** a. Left: Continuous sedimentation distribution obtained for 86 µM DEAF-1 MYND domain. Analysis revealed a 9.95 kDa species suggesting that MYND monomers (5.2 kDa) associate into dimers. The monomer and dimer appear to be in a in rapid monomer-dimer equilibrium (data not shown). Right: Concentration-dependent sedimentation velocity analysis of DEAF-1 MYND domain. Dimerization occurs at sub-micromolar concentrations. The data were fitted to a self-association model yielding an estimated K_D_ of ∼0.5 µM (±0.1 µM). b. Overlay of 1H-15N HSQC spectra recorded at 1 mM (black) and 50 µM (cyan) protein concentration. Cross-peaks that experience line-broadening upon dilution are annotated with the corresponding residue numbers. c. Cartoon representation of the DEAF-1 MYND domain. Residues affected by the dilution of the sample are clustered around the first zinc binding site and are shown as cyan sticks, whereas those involved in binding to co-repressor peptides are shown as magenta sticks. Residue H538 is also shown in black.
(TIF)

Figure S3 **Salt and pH dependance of oligomerization of DEAF-1 MYND domain.** a. Overlay of 1H-15N HSQC spectra (left) and long-range 1H-15N HSQC spectra (right) recorded at a NaCl concentration of 100 mM (black) and 400 mM (green) b. Overlay of 1H-15N HSQC spectra (left) and long-range 1H-15N HSQC spectra (right) recorded at a pH of 6.5 (black) and 6 (red). At pH 6, H538 tends to a conformation where both nitrogens of the side chain, are protonated, thus excluding any possible ion binding. c. 15N R1 and R2 relaxation rates of DEAF-1 MYND domain for three different samples, i.e., [NaCl] = 100 mM and pH = 6.5 (black), [NaCl] = 400 mM and pH = 6.5 (green), [NaCl] = 100 mM and pH = 6 (red). No significant change in relaxation rates are observed changing the buffer conditions. The oligomerization state revealed by the high R2/R1 ratio is thus likely due to non specific interactions.
(TIF)

Figure S4 **Correction of the previously reported structure of DEAF-1 MYND domain.** a. Cartoon representation of the corrected DEAF1-MYND structure with long-range NOEs from the new NMR data. Side chains of residues showing long-range NOEs are shown in as magenta sticks. Newly observed long-range NOEs, which define the cross-brace zinc binding topology, are indicated by green lines. Long range NOEs that were also observed previously are shown as dotted black lines. b. Comparison of original and new NOESY experiments showing important NOEs originating from aromatic residues. Left: The NOE contacts of the His 536 Hε1 proton in a homonuclear 2D NOESY (500 MHz, 120 ms mixing time) and in the 3D aromatic 13C edited NOESY spectrum (800 MHz, 300 ms mixing time). Right: The same for NOEs involving the Tyr 523 Hε1 proton. NOEs observed only with the new experiments are green boxed.
(TIF)

Figure S5 **Binding of E1A to wild-type or mutant BS69 proteins.** The effect of individual mutation of residues RRKR 559–562 of Bs69 for E1A binding was tested. 2× 106 QT6 fibroblasts were transiently transfected with 12SE1A and wt or mutant FLAG-tagged BS69 411–561 peptides as notified on top. 24 h post-transfection, protein lysates were immunoprecipitated with a 10 µg anti-FLAG antibody in a 100 mM NaCl, 20 mM Tris pH8, 0.5% NP40 buffer and Protein A sepharose beads. After extensive washes in the binding buffer, proteins were eluded from beads and separated by SDS-PAGE. Immuno-precipitated E1A protein was revealed by western blotting. Individual mutations of these residues indicate that among these four residues only Arg560 is essential for binding of BS69 to E1A.
(TIF)
