# Supplementary figures and images for: Structural and Functional Analysis of the DEAF-1 and BS69 MYND Domains
Source: PLoS One. 2013 Jan 25;8(1):e54715. doi: 10.1371/journal.pone.0054715 (PMC3555993; doi:10.1371/journal.pone.0054715)

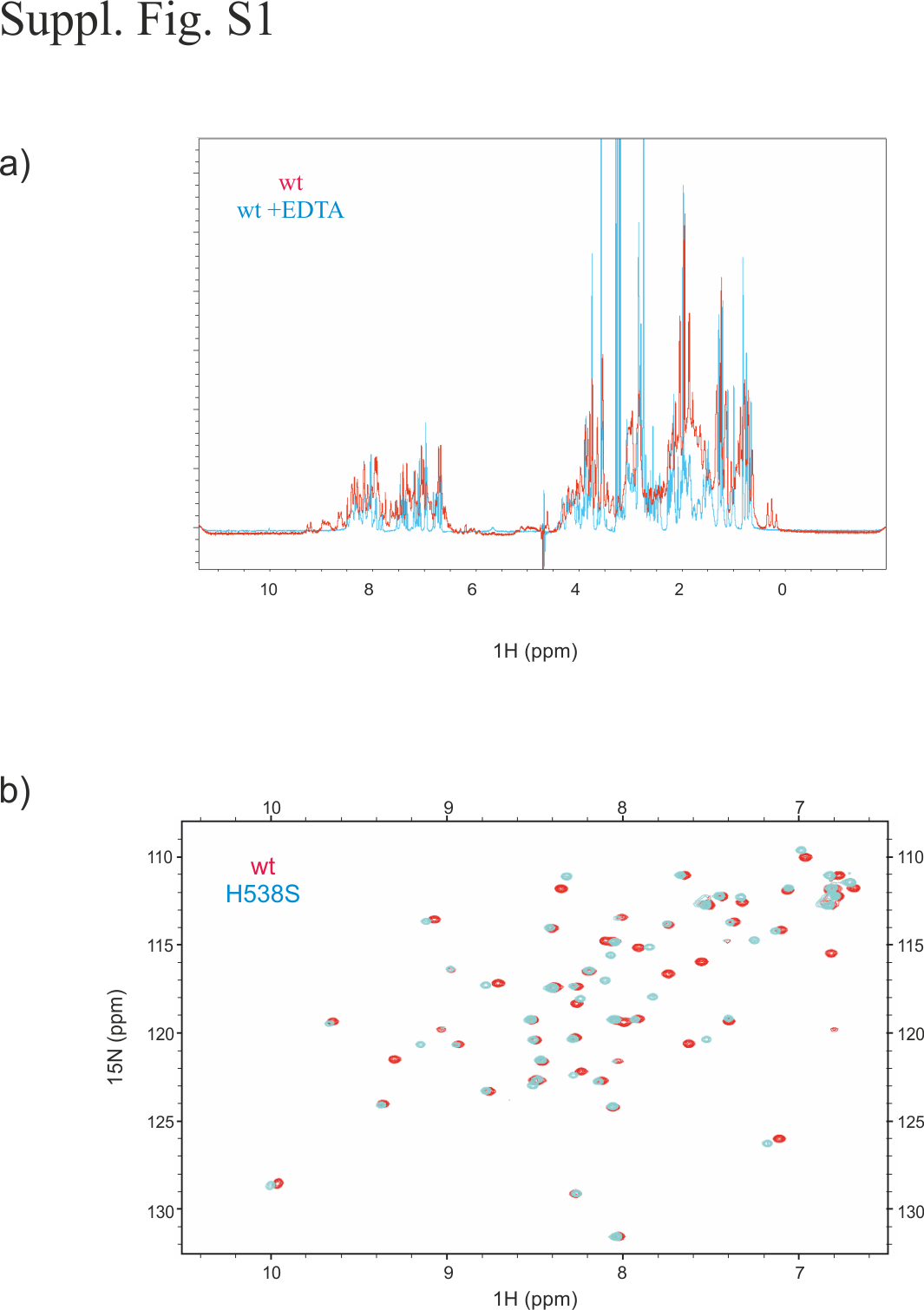

Supplement: Figure S1 — Dependence of the MYND domain fold on zinc coordination. a. Overlay of 1H 1D spectra obtained from the wild type protein in the absence (red) and presence (cyan) of EDTA. b. Overlay of 1H-15N HSQCs of the wild type protein (red) and H538S mutant (cyan). (TIF) [file pone.0054715.s001.tif]

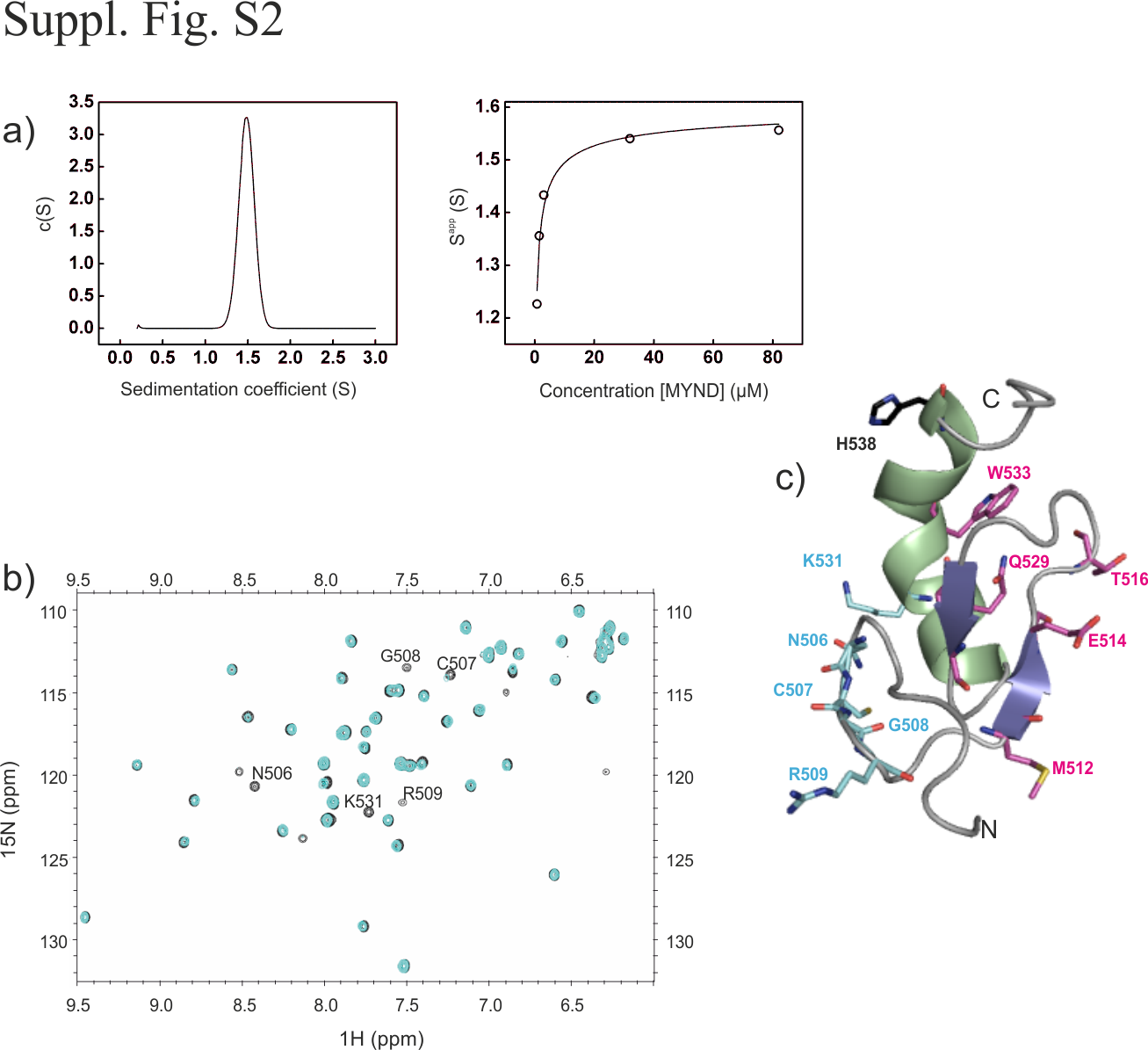

Supplement: Figure S2 — Oligomerization of DEAF-1 MYND domain. a. Left: Continuous sedimentation distribution obtained for 86 µM DEAF-1 MYND domain. Analysis revealed a 9.95 kDa species suggesting that MYND monomers (5.2 kDa) associate into dimers. The monomer and dimer appear to be in a in rapid monomer-dimer equilibrium (data not shown). Right: Concentration-dependent sedimentation velocity analysis of DEAF-1 MYND domain. Dimerization occurs at sub-micromolar concentrations. The data were fitted to a self-association model yielding an estimated KD of ∼0.5 µM (±0.1 µM). b. Overlay of 1H-15N HSQC spectra recorded at 1 mM (black) and 50 µM (cyan) protein concentration. Cross-peaks that experience line-broadening upon dilution are annotated with the corresponding residue numbers. c. Cartoon representation of the DEAF-1 MYND domain. Residues affected by the dilution of the sample are clustered around the first zinc binding site and are shown as cyan sticks, whereas those involved in binding to co-repressor peptides are shown as magenta sticks. Residue H538 is also shown in black. (TIF) [file pone.0054715.s002.tif]

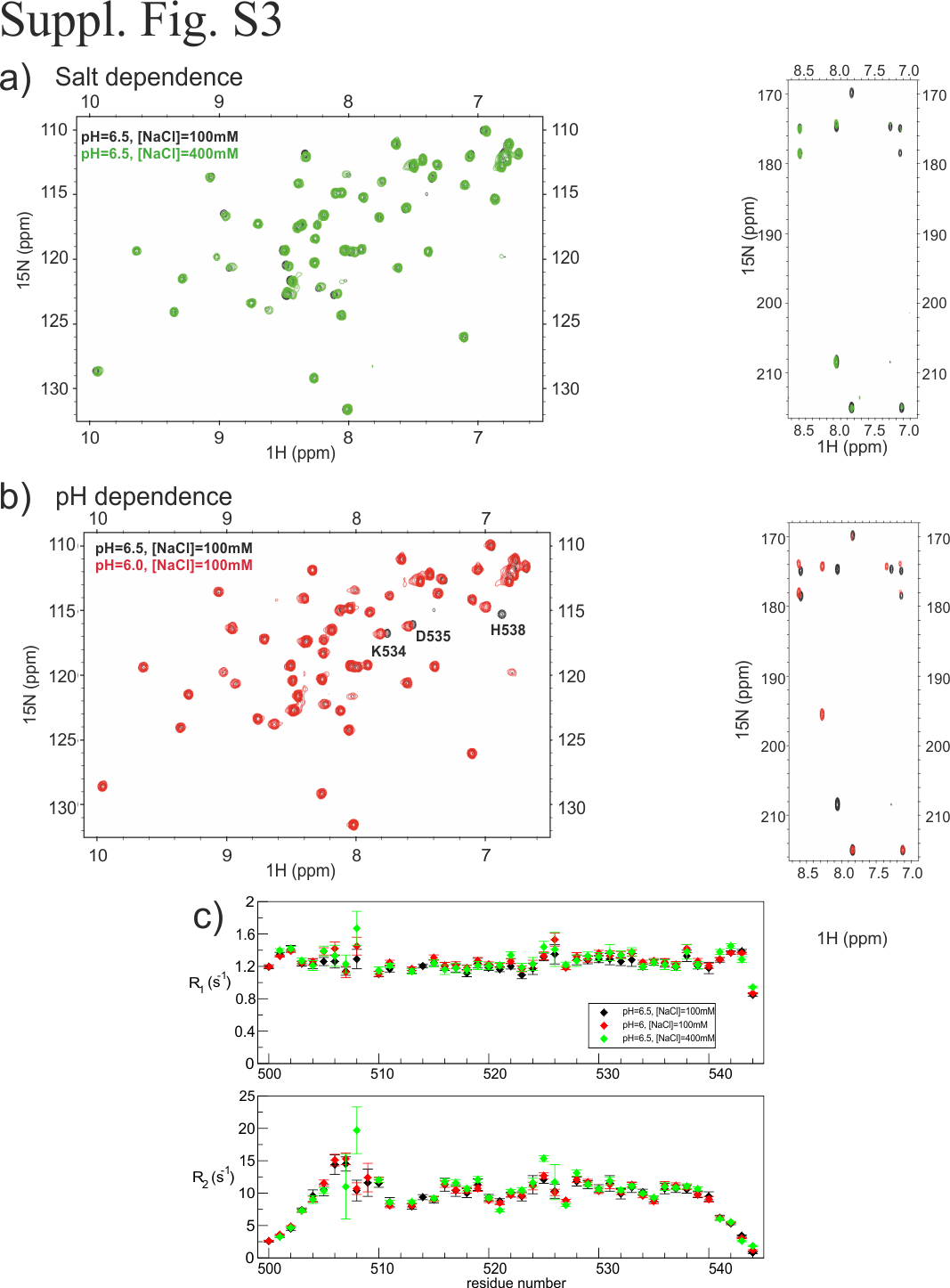

Supplement: Figure S3 — Salt and pH dependance of oligomerization of DEAF-1 MYND domain. a. Overlay of 1H-15N HSQC spectra (left) and long-range 1H-15N HSQC spectra (right) recorded at a NaCl concentration of 100 mM (black) and 400 mM (green) b. Overlay of 1H-15N HSQC spectra (left) and long-range 1H-15N HSQC spectra (right) recorded at a pH of 6.5 (black) and 6 (red). At pH 6, H538 tends to a conformation where both nitrogens of the side chain, are protonated, thus excluding any possible ion binding. c. 15N R1 and R2 relaxation rates of DEAF-1 MYND domain for three different samples, i.e., [NaCl] = 100 mM and pH = 6.5 (black), [NaCl] = 400 mM and pH = 6.5 (green), [NaCl] = 100 mM and pH = 6 (red). No significant change in relaxation rates are observed changing the buffer conditions. The oligomerization state revealed by the high R2/R1 ratio is thus likely due to non specific interactions. (TIF) [file pone.0054715.s003.tif]

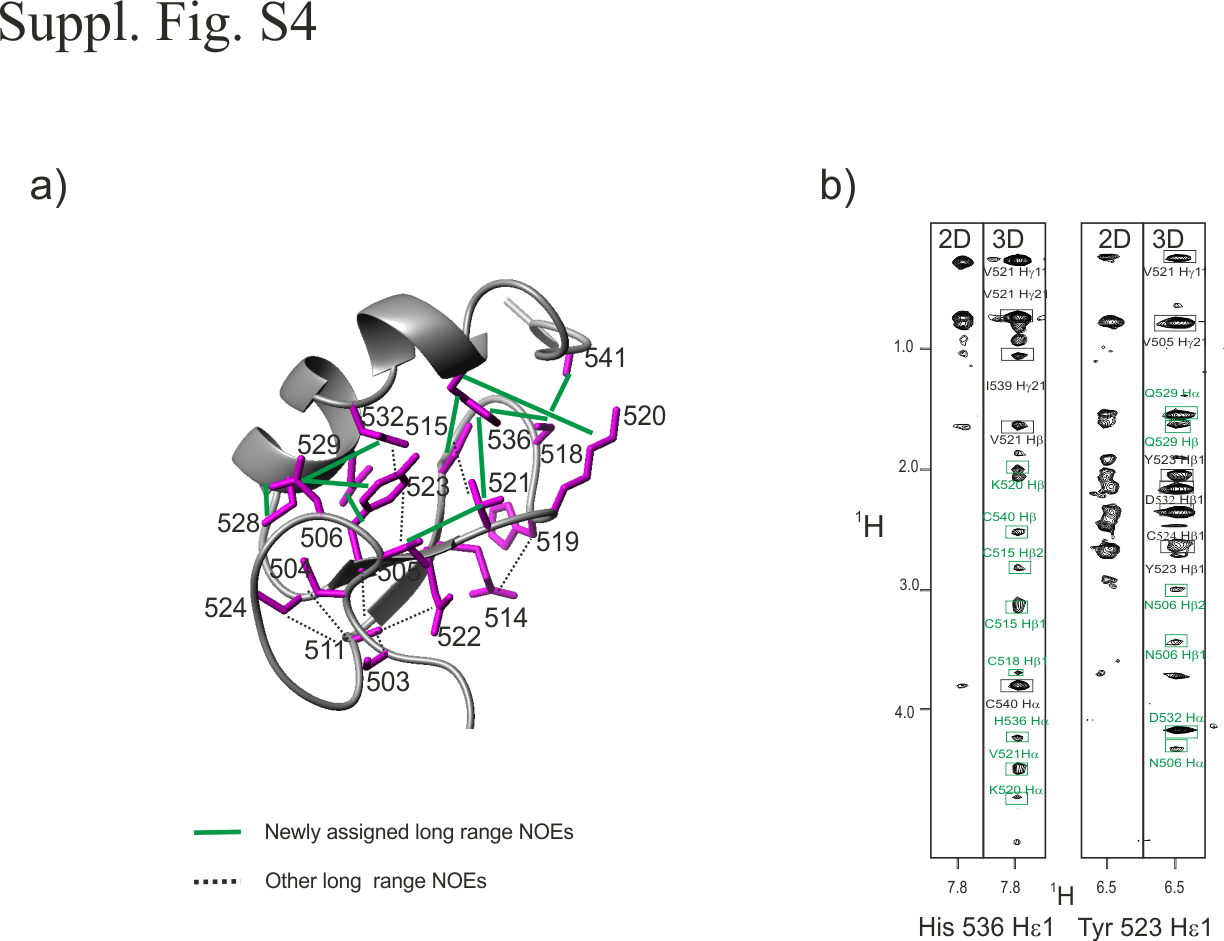

Supplement: Figure S4 — Correction of the previously reported structure of DEAF-1 MYND domain. a. Cartoon representation of the corrected DEAF1-MYND structure with long-range NOEs from the new NMR data. Side chains of residues showing long-range NOEs are shown in as magenta sticks. Newly observed long-range NOEs, which define the cross-brace zinc binding topology, are indicated by green lines. Long range NOEs that were also observed previously are shown as dotted black lines. b. Comparison of original and new NOESY experiments showing important NOEs originating from aromatic residues. Left: The NOE contacts of the His 536 Hε1 proton in a homonuclear 2D NOESY (500 MHz, 120 ms mixing time) and in the 3D aromatic 13C edited NOESY spectrum (800 MHz, 300 ms mixing time). Right: The same for NOEs involving the Tyr 523 Hε1 proton. NOEs observed only with the new experiments are green boxed. (TIF) [file pone.0054715.s004.tif]

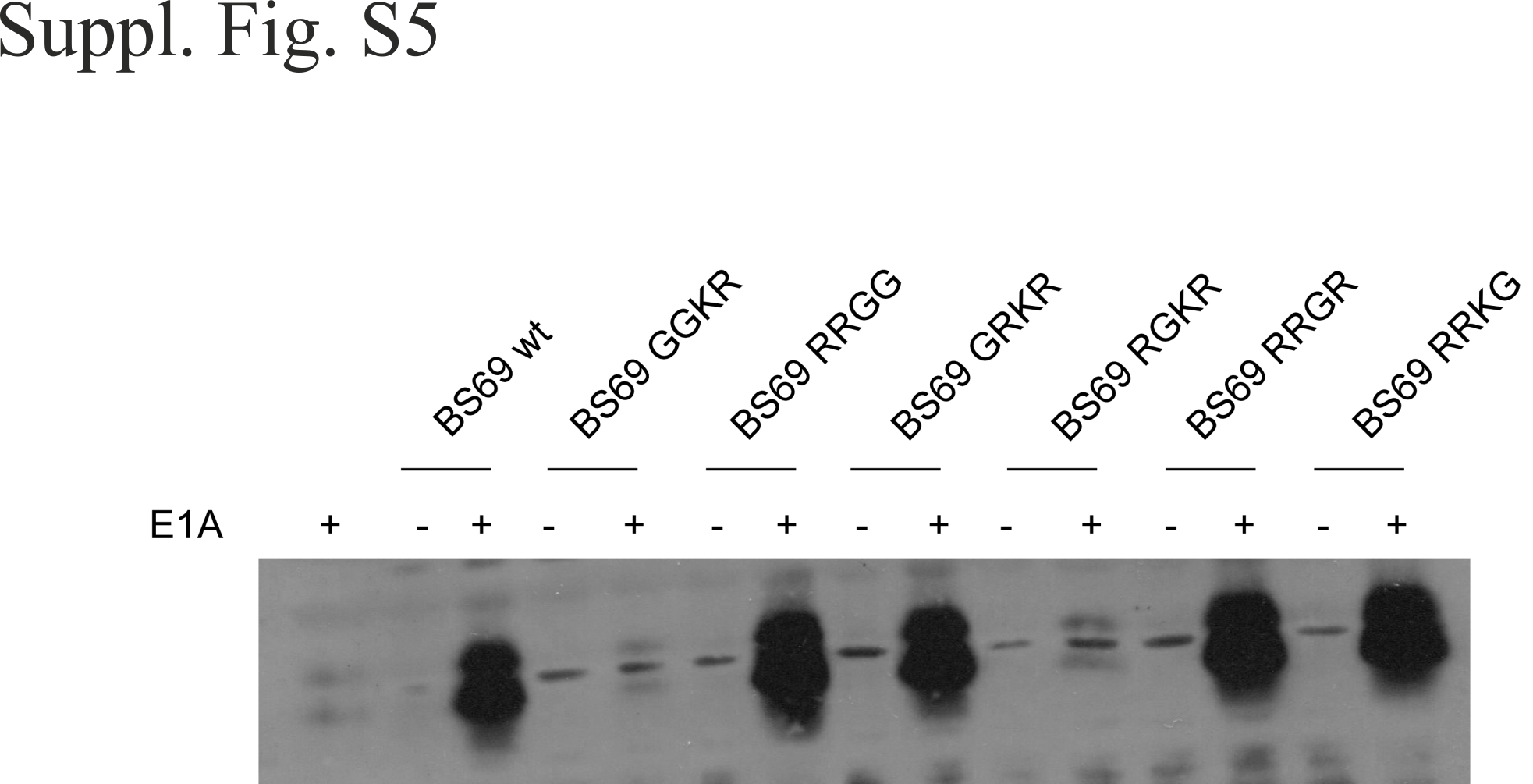

Supplement: Figure S5 — Binding of E1A to wild-type or mutant BS69 proteins. The effect of individual mutation of residues RRKR 559–562 of Bs69 for E1A binding was tested. 2× 10^6^ QT6 fibroblasts were transiently transfected with 12SE1A and wt or mutant FLAG-tagged BS69 411–561 peptides as notified on top. 24 h post-transfection, protein lysates were immunoprecipitated with a 10 µg anti-FLAG antibody in a 100 mM NaCl, 20 mM Tris pH8, 0.5% NP40 buffer and Protein A sepharose beads. After extensive washes in the binding buffer, proteins were eluded from beads and separated by SDS-PAGE. Immuno-precipitated E1A protein was revealed by western blotting. Individual mutations of these residues indicate that among these four residues only Arg560 is essential for binding of BS69 to E1A. (TIF) [file pone.0054715.s005.tif]
